# Learning without forgetting by leveraging transfer learning for detecting COVID-19 infection from CT images

**DOI:** 10.1038/s41598-023-34908-z

**Published:** 2023-05-25

**Authors:** Malliga Subramanian, Veerappampalayam Easwaramoorthy Sathishkumar, Jaehyuk Cho, Kogilavani Shanmugavadivel

**Affiliations:** 1 Department of Computer Science and Engineering, Kongu Engineering College, Perundurai, Erode, Tamil Nadu India; 2 Department of Software Engineering, Jeonbuk National University, Jeongu-si, Republic of Korea

**Keywords:** Health care, Medical research, Engineering

## Abstract

COVID-19, a global pandemic, has killed thousands in the last three years. Pathogenic laboratory testing is the gold standard but has a high false-negative rate, making alternate diagnostic procedures necessary to fight against it. Computer Tomography (CT) scans help diagnose and monitor COVID-19, especially in severe cases. But, visual inspection of CT images takes time and effort. In this study, we employ Convolution Neural Network (CNN) to detect coronavirus infection from CT images. The proposed study utilized transfer learning on the three pre-trained deep CNN models, namely VGG-16, ResNet, and wide ResNet, to diagnose and detect COVID-19 infection from the CT images. However, when the pre-trained models are retrained, the model suffers the generalization capability to categorize the data in the original datasets. The novel aspect of this work is the integration of deep CNN architectures with Learning without Forgetting (LwF) to enhance the model’s generalization capabilities on both trained and new data samples. The LwF makes the network use its learning capabilities in training on the new dataset while preserving the original competencies. The deep CNN models with the LwF model are evaluated on original images and CT scans of individuals infected with Delta-variant of the SARS-CoV-2 virus. The experimental results show that of the three fine-tuned CNN models with the LwF method, the wide ResNet model’s performance is superior and effective in classifying original and delta-variant datasets with an accuracy of 93.08% and 92.32%, respectively.

## Introduction

COVID-19 is a contagious disease-causing respiratory illness that affects the lungs and respiratory system after exposure to the virus. The earlier symptoms include cold, fever, and difficulty in breathing may appear between day two to fourteen. The condition of the infected person may have mild to severe effects depending on immune strength and disease severity. A person infected with the coronavirus may be contagious for up to two days prior to the onset of symptoms and 10 to 20 days following the onset of symptoms. The Covid-19 outbreak is currently spreading over the globe. Currently, the Reverse Transcription Polymerase Chain Reaction (RT-PCR) is used to identify COVID-19 using a laboratory examination of samples from the nose and throat for a formal diagnosis of Covid-19^1^. However, the RT-PCR procedure requires specialized test kits, which may have been in short supply and necessitate a minimum of 24 h. Due to the limited availability of COVID-19 test kits, an alternative method of utilizing radiological images is effective for diagnosing the infection. Chest imaging can be used to diagnose individuals with Covid-19 symptoms while waiting for RT-PCR given the individual’s Covid-19 symptoms. A study from China shows that chest Radiography and Computed Tomography (CT) scans can help identify the infection. The images are used to signal abnormalities in lung infection, particularly Covid-19. In the early identification of Covid-19 illness, chest CT is superior to chest X-ray. Therefore, chest CT scans are ideal for diagnosing lung-related issues. A chest CT, a diagnostic tool for pneumonia, can be performed rapidly and with considerable ease. Compared to RT-PCR, multiple studies have found that CT scans have more excellent diagnostic performance because they can identify disease-related lung patterns, like ground-glass opacities. As a result, CT has become one of the essential methods for earlier detecting and diagnosing Covid-19^1,2^. However, chest CT scans, on the other hand, can produce both false-negative and false-positive results. Therefore, better computer-assisted CT lung diagnosis procedures are crucial to effectively confirm suspected infections, screen patients, and undertake viral monitoring. As AI has progressed, image-identification-focused computer vision algorithms have been applied to medical imaging, including CT images^3,4^. Several researches have used medical imaging-based deep learning systems to develop models to extract image information, including textural extraction and spatial correlations. CNNs excelled in various computer vision tasks^4,5^. CNN produced very impressive results in the extraction of visual characteristics. Due to their self-learning capabilities, CNNs have recently become the primary deep learning approach for medical image classification. Numerous neural network models based on CNN for Covid-19 detection have been recommended^6^, and these algorithms require relatively few medical image datasets for training. Meanwhile, studies related to parameter tuning, data enrichment, and transfer learning have all been employed by these CNNs to attain new levels of efficiency. This research aims to provide effective methods for utilizing CNN to detect the infection from CT images.

## Research focus

During the preliminary work and background study, this research identifies the potential for deep learning models to improve image characteristics through AI and provide more reliable imaging techniques to detect viral infection. This work postulates that AI techniques could extract particular graphic aspects of COVID-19 and provide a clinical diagnosis before the pathogenic test, thus saving critical time for disease control. Hence, we plan to focus on AI-based deep learning models to detect Covid-19 from CT images. We intend to answer the following research questions with this study:

- *RQ1* How can deep learning models that have been pre-trained be customized?
  We fine-tuned the top layers of pre-trained models to classify the images under consideration.
- *RQ2* How effective can fine-tuning the hyperparameters of different CNN models be?
  Since the values of hyperparameters are so crucial for judging how well a model works, we used Bayesian Optimization to figure out the best values that should be used for model evaluation.
- *RQ3* Can models use what they’ve learned from one task or dataset to do something new and excel in original and new datasets?

To analyze the learning capability, we have evaluated the performance of the models with a set of CT scan images belonging to the Delta variant, a type of coronavirus COVID-19. In this study, we develop a collection of models employing pre-trained CNN architectures to automatically detect and characterize Covid-19 infection using CT images acquired from diverse sources such as Kaggle, etc. To fine-tune pre-trained models for repurposing, we may employ two strategies: feature extractor and fine-tuner. Since it is found that the performance could be better when the pre-trained models are deployed as feature extractors without fine-tuning, we plan to employ fine-tuning^7^. In fine-tuning, the top convolution layers of pre-trained models are retrained. This work also uses Bayesian optimization to find the ideal configuration for hyper-parameters utilized for training different CNN architectures. The following are the substantial contributions to this work:

1. Investigation of the performance of CNN variations using transfer learning, fine-tuning, and detailed simulations.
2. Exploration of Bayesian optimization to hyper-parameter optimization
3. Transferring the knowledge acquired by the proposed models to a standard, but challenging dataset comprising scans of the Delta variant of Coronavirus

We analyze the performance of three CNN architectures using transfer learning over the CT image dataset. Learning without Forgetting (LwF) is investigated as a solution for multitask learning. Since Bayesian Optimization uses information from past models to choose the hyperparameter values for a new model, it takes less time than the other methods to get to the most accurate model^8^. To the best of our knowledge, no previous work has focused on transfer learning combined with fine-tuning approaches optimized with Bayesian Optimization to classify COVID-19 infection. In addition, the LwF is attempted and included as a classification solution for a new task in classifying COVID-19. As far as we know, the proposed study is the first work in the literature that combines CNN with transfer learning and LwF technique in the field of CT scan image classification for detecting COVID-19, which is the primary contribution of the paper.

The remainder of the article is organized as follows: In “Literature survey” section, it is addressed how current research has attempted to use CNN-based deep learning algorithms to detect Covid-19 infection from CT images. “Materials and methods” section discusses datasets, deep neural network topologies, fine-tuning techniques, Bayesian optimization, and LwF in depth. Experimental setup and hyper-parameter tuning are discussed in “Experiments” section. “Results and discussion” section highlights the results of the experiments and provides a comparison of the proposed models. “Findings and discussion” section provides the experimental results, findings from the proposed work, and error analysis. Finally, the summary of our work and conclusion are provided in “Conclusion” section.

## Literature survey

This section presents an overview of the recent deep learning models that help to detect Covid-19 infection from CT images.

Deep CNN-based approaches for detecting Covid19 in CT images have been reviewed by Mishra et al.^9^. A decision fusion approach is also put forth, which combines forecasts from many models to arrive at a final prediction. According to the findings of this approach, the proposed technique based on decision fusion can obtain over 86% on all performance metrics. A deep learning algorithm developed by the authors in^10^, Chest CT scans are used to separate and measure infection regions and the entire lungs automatically. In this study, the authors developed VB-Net to segment Covid-19 infection spots in CT images. 249 Covid-19 CT scans were used for the segmentation technique. Wang et al.^11^ have investigated the detection of COVID-19^9^ and developed an algorithm by modifying the inception transfer-learning model and then testing it internally and outside. Hasan et al.^6^ employed DenseNet-121 to identify patients with Covid-19 by testing on many datasets. In an attempt by Song et al.^12^, chest CT images were gathered from 88 Covid-19 patients from hospitals in two Chinese regions, which also contain data of 100 pneumonia patients, and 86 healthy people for comparison and modelling. Based on this data, a deep CT diagnostic method for identifying Covid-19 patients has been built. This work claimed that the proposed models could effectively identify Covid-19 patients. In an attempt by Narin et al.^13^, patients with a coronavirus pulmonary infection were determined using a set of five CNN-based models that were pre-trained. Three distinct binary classifications, with four classes, have been constructed using five-fold cross-validation. The pre-trained ResNet50 model appears to have the highest classification accuracy, as the findings indicate. Singh et al.^14^ used a CNN to determine whether or not individuals have been infected with the COVID-19 virus. Additionally, to fine-tune the initial parameters of CNN, the multi-objective differential evolution method is utilized. This work carried out extensive research using proposed and competing machine learning techniques to be capable of accurately classifying chest CT scans.

Deep transfer learning models were used by Rezaeijo et al.^15^ to propose a method for predicting COVID-19 disease from chest CT scans. Machine learning algorithms such as RF, SVM, DT, KNN, and LGR have been used with deep learning models like ResNet, Xception, DenseNet, and VGG16 for predicting the SARS-CoV-2 infection. Zhao et al.^16^ tested a CNN and pre-trained CNN models for COVID-19 using CT scans. They conducted transfer learning on CT images used for COVID-19 testing and evaluated the effects of various starting settings, revealing that the ImageNet-trained model proposed has good CT generalizability. Several hybrid learning models were employed in^17^ to categorize COVID-19 CT images, and the results of this classification were examined for improved data analysis. Roberts et al.^18^ performed a comprehensive analysis of the published articles and preprints explaining novel machine learning models used to diagnose or predict the prognosis of COVID-19 based on CT scans. This work also addressed the shortcomings of the previous works and provided recommendations for the construction of models of high quality. In addition to the methods described above, other models have also been devised to identify cases of Covid-19 infection based on CT scans. ElAraby et al.^19^ developed a unique Gray-Scale Spatial Exploitation Net (GSEN) to quickly identify infected COVID-19 instances by collecting COVID-19 photos from various websites using a web crawler-based cloud environment. Using Stochastic Gradient Descent (SGD) Optimizer, the hyperparameters of GSEN were optimized in this work.

A lightweight CNN model called LightEfficientNetV2 was proposed by Huang and Liao^20^ for the detection of COVID-19, Pneumonia, and Normal using X-ray and CT scans with a limited number of images. This study examined and provided the results of transfer learning models employed before and after fine-tuning. Gour and Jain^21^ built a stacked convolutional neural network model for automatic COVID-19 identification from chest X-ray and CT images. During training, this method obtains several sub-models from the VGG19 and Xception models and then evaluates how well those models perform. Using a pre-trained VGG19 deep CNN architecture and the YOLOv3 detection algorithm, Karachi^22^ sought to classify COVID-19 through X-ray images with good precision ratios. To accomplish this, the authors developed the VGG19, VGGCOV19-NET, and original Cascade models and fed them data using the YOLOv3 algorithm. Joshi et al.^23^ propose a lightweight multi-scale CNN called LiMS-Net, which consists of two feature learning blocks in which, in each block, filters of different sizes have been used in parallel to derive multi-scale features from the suspicious regions, and an additional filter is then used to capture discriminant features. Extensive experiments on the publicly available COVID-19 CT dataset show that the proposed model outperforms many pretrained CNN models and state-of-the-art methods despite the scarcity of CT data. Hassan et al.^24^ provided a thorough analysis of how deep learning models can be used for identifying Covid-19 and segmenting the lungs. Feature extraction from CT and X-ray images was the focus of this study. The difficulties of using deep learning strategies have also been covered. Elzeki et al.^25^ proposed a Nonsubsampled Contourlet Transform to break the image into subbands. CNN-VGG19 is then used for feature extraction from COVID-19 images. The findings showed that the pre-trained framework employing deep learning VGG19 fusion might significantly affect the classification and feature extraction from X-ray COVID-19 images automatically related to the diagnosis of COVID-19. In an effort by Elzeki et al.^26^, a model called Chest X-Ray COVID Network (CXRVN) is proposed for analyzing and evaluating grayscale CXR images based on three different COVID-19 X-Ray datasets. CXRVN model is a lightweight architecture that uses a single fully connected layer to represent the essential features. This reduces the total amount of memory and the time it takes to process compared to pre-trained and other models. A summary of the literary works is presented in Table 1.

**Table 1:** Summary of literature survey.

Reference	Algorithms	Performance metrics	Datasets
^9^	Deep CNN : VGG16, Inception V3, ResNet50, DenseNet121, DenseNet201	AUC-ROC—0.883 F1-Score—0.867	medRxiv and bioRxiv
^10^	DL-based segmentation system	Dice similarity coefficient—91.6% ± 10.0% Accuracy of severity prediction—73.4% ± 1.3%	–
^11^	inception transfer-learning model	Accuracy—89.5% Specificity—0.88 Sensitivity—0.87	Collected from web resources
^12^	ResNet based DRENet	AUC-ROC—0.95, Recall—0.96, Precision—0.79	From the hospitals of two provinces in China
^13^	ResNet50, ResNet101, ResNet152, InceptionV3 and Inception-ResNetV2)	96.1% accuracy for Dataset-1, 99.5% accuracy for Dataset-2 and 99.7% accuracy for Dataset-3	GitHub and Kaggle repository ( 3 datasets)
^14^	CNN, ANN, and ANFIS	Accuracy—more than 93%	–
^15^	CNN and Deep transfer learning and machine learning models	Accuracy—100%	Web resources
^16^	Deep convolutional neural network	Positive Predictive Value—98.5%, Specificity 99.5%, Negative Predictive Value—99.6%	Healthcare institutes from China and Iran
^20^	InceptionV3, ResNet50V2, Xception, DenseNet121, MobileNetV2, EfficientNet-B0, and EfficientNetV	Accuracy—96.50% (InceptionV3)	Kaggle and Mendeley dataset
^21^	Stacked CNN	Sensitivity—97.62%	GitHub and Mendeley
^26^	Deep learning based X-ray COVID-19 Network architecture	Accuracy—94.5%	Kaggle website
^25^	NSCT + CNN_VGG19	Q^AB/F^, Q^MI^, PSNR, SSIM, SF, and STD	Images acquired from 25 cases
^27^	InceptionV3, AlexNet, DenseNet121, VGG19, and MobileNetV2	Precision—96.71%, Recall—96.83%, Specificity—99.78%	Kaggle and other web resources

We conclude from recent studies that deep learning models are increasingly used to identify Covid-19 infection using CT scans. However, a few areas of improvement need to be made in applying deep learning architectures, including quicker training times and better tuning of hyper-parameters. Therefore, our motivation is to build a few classification models that can discriminate between Covid infected and not-infected images using a combination of neural network techniques (such as deep learning and transfer learning) with hyperparameter tuning to improve the performance and learning ability of the model. Further, we have used the LwF technique to retain the original network capabilities while training the models on a new dataset.

## Materials and methods

The primary objective of this research is to construct and compare a few neural network models for classifying CT scans using different CNN architectures. In addition, traditional and modern CNN architectures will be used to evaluate their performance and identify models that can accurately diagnose Covid-19 infection. This section describes the materials and methods utilized for this investigation.

### Dataset

**Figure 1:**
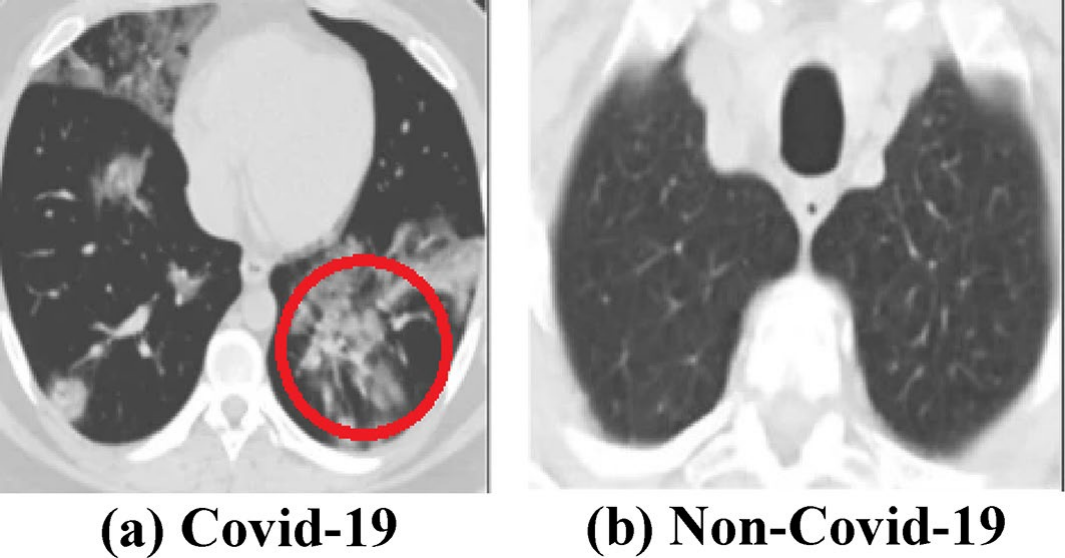
Covid and non-covid samples (https://figshare.com/articles/dataset/COVID-19_Chest_X-Ray_Image_Repository/12580328).

All stages of object recognition necessitate a sufficient dataset. Covid-19 and non-Covid-19 CT images have been gathered from various web sources such as Kaggle^28^, Google^29^ and other web resources^30,31^ and categorized into two groups. The CT images depicted in Fig. 1 are examples of each class namely Covid-19 in Fig. 1a and normal image in Fig. 1b, respectively^18^. The red circle in Fig. 1a designates the lung infection showing the area affected by Covid-19. In this dataset, we have 980 images; 459 images have Covid-19 infection and 521 images belong to normal or unaffected images, respectively.

### Image augmentation

A neural network needs a lot of data to train the model to learn more features. We have used image augmentation techniques to include more images into the dataset with different orientations and shapes as we only had a few images to work with. Image augmentation is an excellent way to train deep learning models when we don’t have enough data. To increase the number of images through the image augmentation technique, we have applied operations like rotation, shifting, zooming, mirroring and flipping as some of the image-enhancing techniques along with blurring, scaling, cropping and padding techniques^27,32^. In this study, we have applied image augmentation techniques based on “in-place” and “on-the-fly” methods. Real-time enhancements are the primary benefit of implementing in-place augmentation. In several ways, augmented images can be created in real-time while the model is still being trained. However, the images in the dataset are pre-processed to make it easier to find features before the training process. After performing pre-processing and image augmentation, we finally have 3562 images in our dataset for training the neural network models. The dataset has been split into 85% training, and 15% test sets to test the different CNN architectures. The training dataset is used for fivefold cross-validation.

### Methods

The development of several fundamental CNN architectures for image recognition applications has resulted in their effective application to various complex visual imagery challenges. In this study, the ability of three different CNN architectures, namely VGG16, ResNet and wide ResNet, to detect infection in CT scans was evaluated to identify COVID-19 infection. These models are summarized in the following sections.

#### Basic CNN

**Figure 2:**
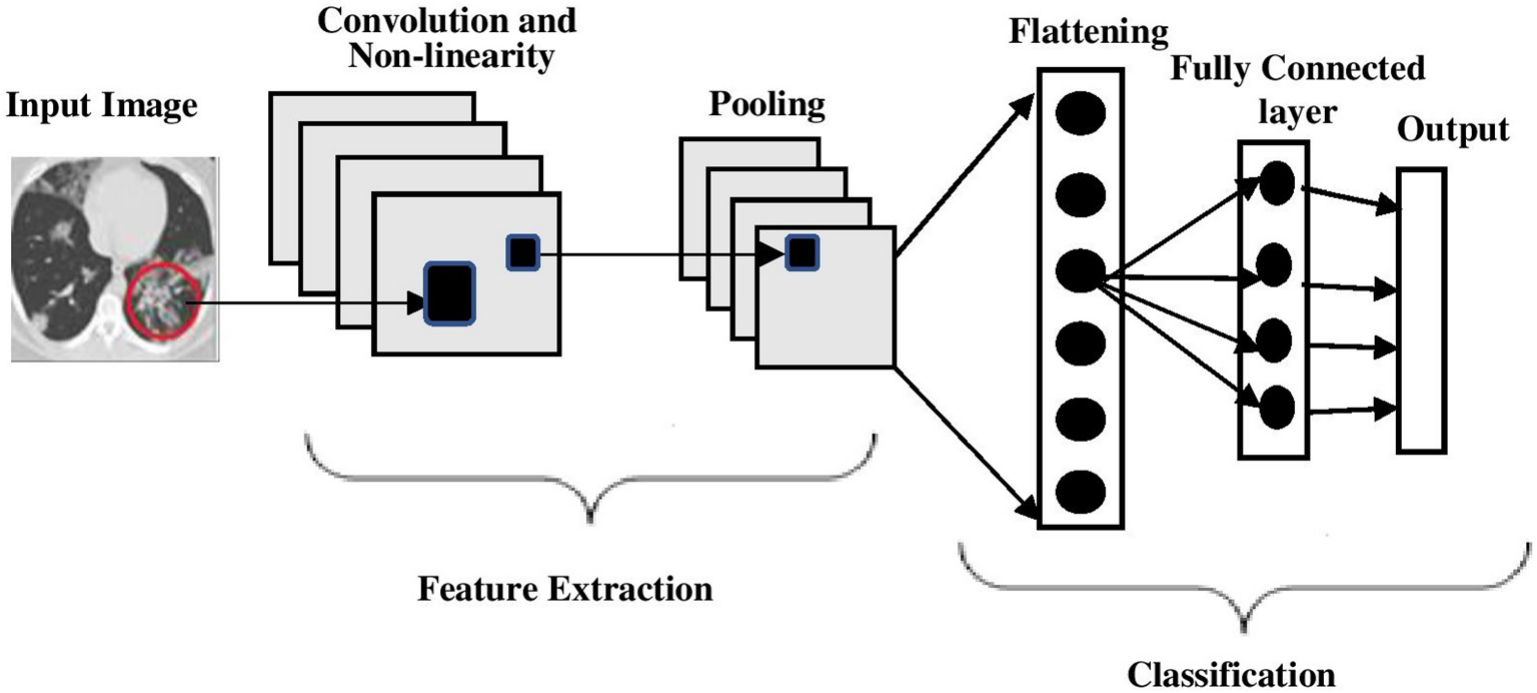
Architecture of CNN.

CNN architecture is a widely used deep learning tool for categorizing images, and it is also a promising approach for modelling applications that use a lot of data^33,34^. Using CNN with a gradient-based method, Lecun et al.^35^ successfully tackled the problem of classifying handwritten digits. Figure 2 depicts the typical architecture of a CNN. CNN has the key capability of automatically learning hierarchical feature representations. The initial few layers of a CNN are often responsible for detecting fundamental properties such as horizontal, vertical, and diagonal edges, among others. The output of these layers is sent to the intermediate layers, which are responsible for extracting more complex features, such as corners and edges. As we move deeper into the network, the layers begin to identify higher-level items such as objects, faces, and other similar things. CNN substantially benefits conventional feed-forward neural networks in that it requires a reduced number of neurons and hyper-parameters. In this study, we develop the proposed models using pre-trained CNN models such as VGG16, ResNet and Wide ResNet.

#### CNN variants

The development of several fundamental CNN architectures for image recognition applications has resulted in their practical application to various complex visual imagery challenges. CNN architecture is a widely used deep learning tool for categorizing images, and it is also a promising approach for modeling applications that use a lot of data^33–35^. Using CNN in conjunction with a gradient-based method, Lecun et al.^35^ successfully tackled the problem of classifying handwritten digits. In this study, we develop the proposed models using pre-trained models such as VGG16, ResNet and Wide ResNet. The rationale behind using these pre-trained models is outlined below.

VGG16 has up to 16 layers and is developed as a deep CNN^36^. This model outperforms ImageNet in a range of tasks and datasets. Since VGG16 is still one of the most prominent image-recognition algorithms, we plan to employ it. Heet al. proposed ResNet, which is seen as a new development of deep networks^37^. Through the implementation of residual learning in CNNs, ResNet sparked a revolution in CNN architectural race^38^. He et al.^37^ empirically demonstrated that ResNet with 50/101/152 layers has fewer errors while doing image classification. The feature reuse problem, in which some feature changes may contribute relatively little to learning^38^, is the fundamental shortcoming of deep residual networks. Wide ResNet^39^ addressed this issue. Zagoruyko et al.^39^ presented a new, widened architecture for ResNet blocks that, in addition to enabling residual networks with much-enhanced performance, also allows residual networks to be constructed. By making ResNet wide rather than deep, Wide ResNet could take advantage of the strength of the residual blocks^37^. Wide ResNet showed that increasing the layer width may be a more efficient strategy to enhance performance than increasing the depth of the residual networks.

#### Transfer learning

In recent years, researchers have increasingly used advanced methodologies for improving the performance of the model and, in particular, become interested in transfer learning. Transfer Learning develops new models of AI by refining previously trained neural networks^40^. Typically, it is depicted using pre-trained models in computer vision applications. Using the ImageNet database, Canziani et al.^41^ evaluated the performance of pre-trained computer vision models. As depicted in Fig. 2, a CNN comprises two distinct components: a convolutional base and a classifier. The fundamental function of the convolutional base is to extract image features for the classification process. Using the extracted features, the classifier can classify the image. The basic features are learned at the bottom of a CNN’s convolutional base; specialized features are learned at higher levels.

Especially when we have a limited sample size, which is usual in medical imaging, ImageNet-based transfer learning is advantageous, as studied in^42^. In this work, the early layers of the pre-trained model are frozen, and only the remaining higher-level layers are retrained. Upper layers must be retrained to account for the new data. In such a case, we employ the pre-trained model as a classifier. However, due to the low similarity between the new dataset and ImageNet, it is necessary to retrain and modify the top layers to consider the different variations in the new data. For retraining, we unfreeze a few top layers of the pretrained models. The pre-trained models have 1000 distinct labels at the last layer. But, for our work, the number of labels is the number of distinct classes in the dataset, that is, two classes. We may therefore import the convolution base and include our classifier for the classification of the COVID-19 disease, and then the classifier is fed with the output of the convolution base.

### Learning without forgetting

**Figure 3:**
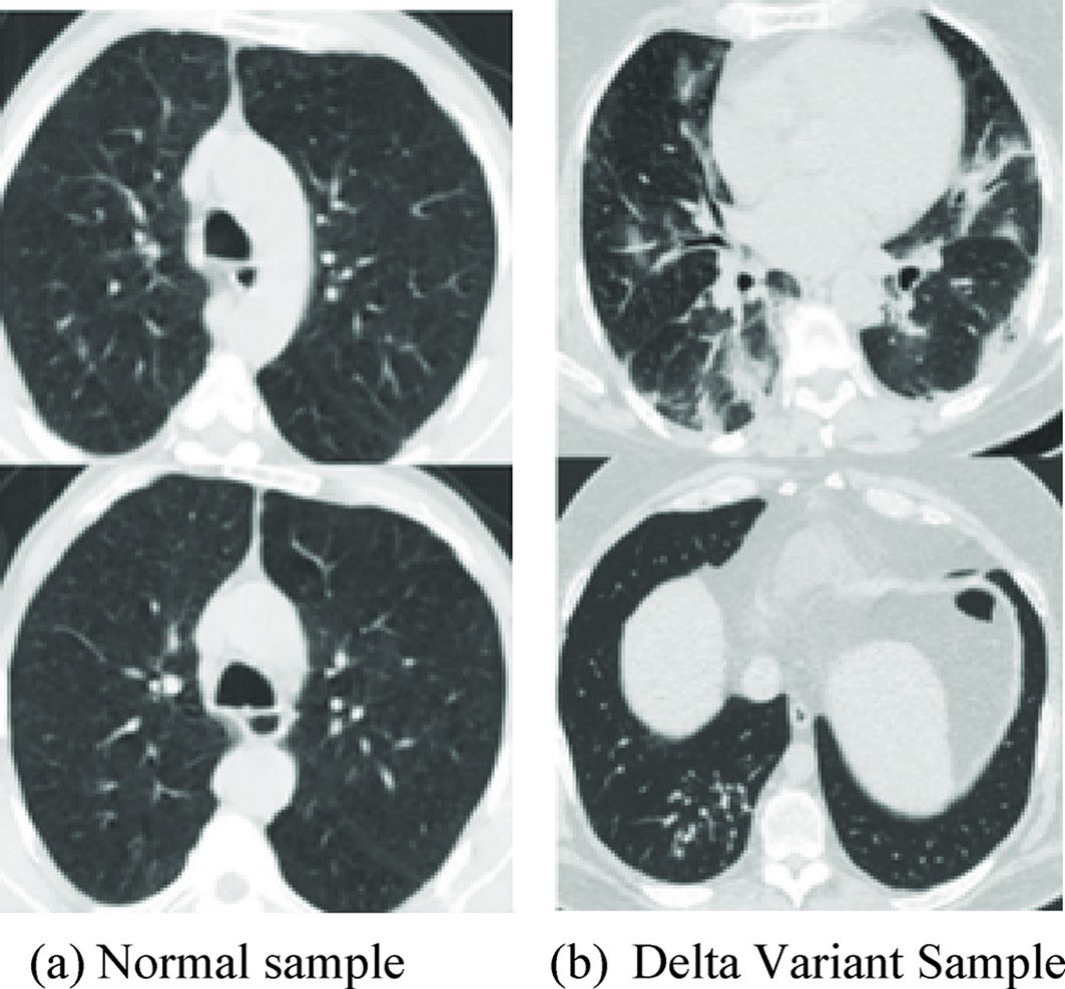
CT images for Normal and Delta variant samples.

The deep neural network model does not need feature engineering to extract or generate features from the data. However, the shared parameters must effectively reflect what makes the new task different; feature extraction usually only works well when applied to the new task. When a model is retrained on a new dataset, it may lose the parameters specific to the original tasks and can no longer do well on the original tasks. Even though transfer learning was created to use the knowledge of a pre-trained model trained on a large enough database to solve other relevant problems, it needs to consider how well the model performed on the previous tasks. A pre-trained CNN might forget what it learned when the knowledge is transferred to another task. For example, suppose a CNN classifier that had already been trained to classify the types of cars is used to classify car genres using transfer learning. In that case, it works well at recognizing car genres but not so well at categorizing the types of cars. In such a situation, transfer learning needs to be improved during the evaluation. To overcome this limitation while applying transfer learning, a concept of Learning without Forgetting (LwF)^43^ has been suggested as a solution to this problem. It works well on the new tasks while keeping the same performance on the old ones. LwF aims to teach a network how to do well on old and new tasks when only data from new tasks are available. With this strategy, the responses from the old network are kept, and samples from the new task are used to improve the accuracy of the new task. Moreover, this method does not need the images and labels from the old task. Hence, as an attempt, we have utilized CT images that show the symptoms of the Delta variant in the CNN model incorporated with LwF to figure out how they are performing on the new dataset. Figure 3 shows a few CT images that belong to normal and delta-variant infected cases.

### Bayesian optimization

**Figure 4:**
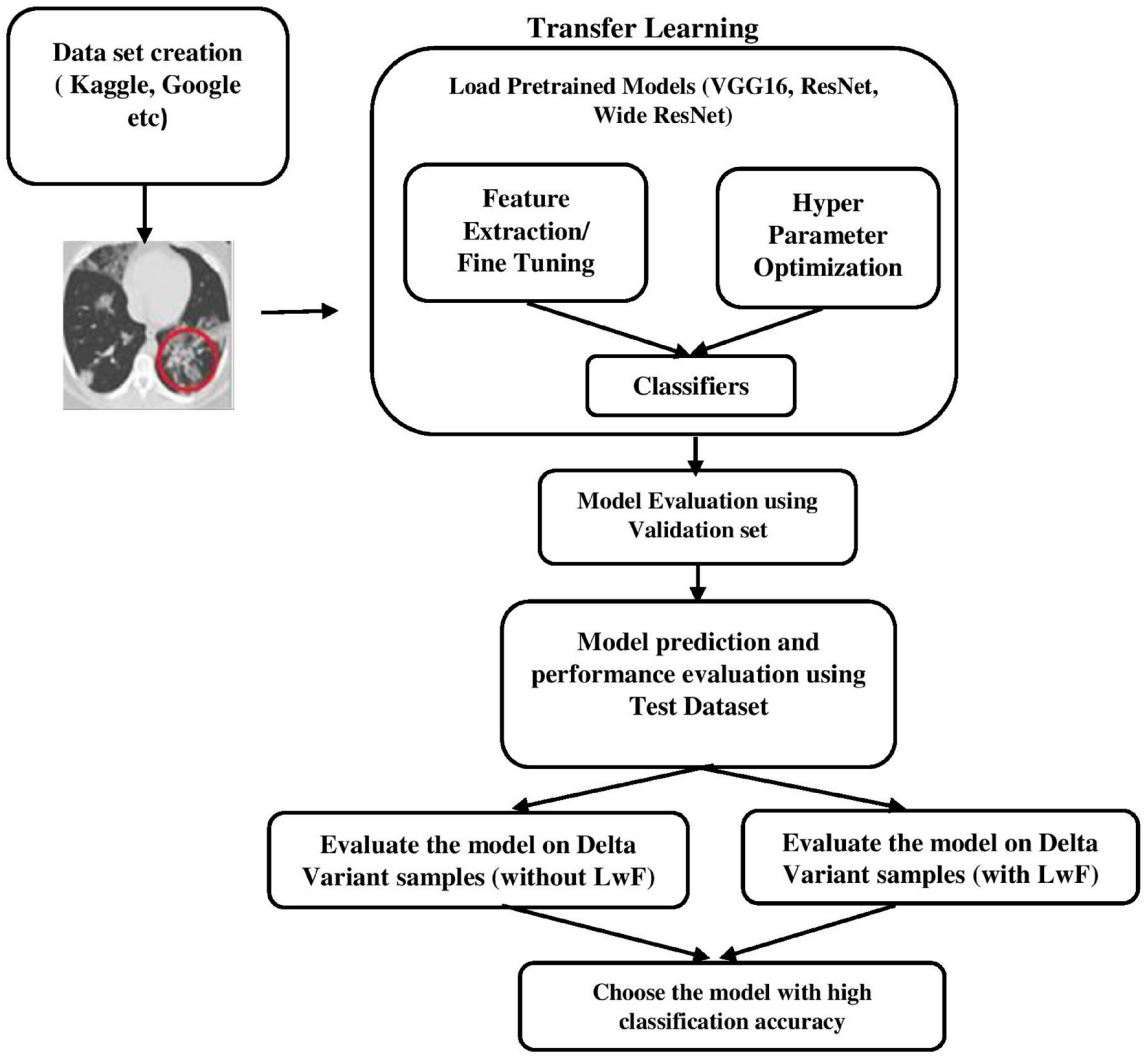
Proposed workflow.

The performance of the deep neural network model dramatically depends on the selection of optimal hyper-parameters during model development and validation. The objective of hyper-parameter tuning is to determine the optimal settings of hyper-parameters so that a loss function can be minimized and the results can be improved. Bayesian optimization brings us closer to the optimal solution with each alternate hyperparameter selection for a test. In other words, Bayesian optimization facilitates the selection of the optimal combination of hyper-parameters to evaluate a model^8,44–46^. Training deep learning models can be time-consuming because of the volume of data involved and the complexity of the computations required. When faced with problems of this nature, applying Bayesian optimization can significantly improve the model performance in less computational time. Figure 4 illustrates the proposed architecture that depicts the research workflow.

## Experiments

This section discusses the sets of experiments conducted using fine-tuned pretrained models with LwF.

### Experimental platform

The execution of the deep learning models requires high computational resources, high-power consumption, and high-performance hardware, so we used Graphical Processing Units (GPU) to run the proposed models. We imported the appropriate Keras model architectures and instantiated them using the ImageNet dataset.

### Tuning of hyper-parameters

When using deep learning algorithms, hyper-parameters define training parameters and influence the model output. The Bayesian optimization approach can be used to determine an objective function’s minimum or maximum value. Optimizers, learning rate, activation function, number of epochs, batch size, and the number of neurons are among the hyperparameters that have been fine-tuned. The hyperparameters used in our proposed work are summarized in Table 2, along with their experimental ranges in the search space.

**Table 2:** Hyper-parameters and their search space.

Parameter	Search space
Optimizer	RMSProp, ADAM, SGD
Learning rate	1e−2, 1e−3, 1e−4, 1e−5, 1e−6
Activation function	Relu, Elu, LeakyRelu, and Tanh
Number of neurons in customized layers	32, 64,128, 256, 512,1024
Number of epochs	50,75,90, 100
Batch Size	16,32,64

### Experimental setup

While testing, we retrained some of the higher layers of the convolution bases of the proposed models for improved performance. However, because the initial layers learn lower-level features from the input images, we do not need to retrain them. In addition, Bayesian optimization is employed to determine the optimal hyperparameter values. The Bayesian optimization procedure has been run fifty times. Each cycle of Bayesian optimization has 100 epochs. We tracked how well we did and how much we lost during the evaluation process at each step. Table 3 shows the values of the hyperparameters that gave each strategy the most superior results across all models. Since more iterations did not have much impact on performance, the hyper-parameters found at 100 iterations were considered in our study. However, we might get a better set of hyper-parameter values if we use an enormous search space, but it will take longer.

**Table 3:** Hyper-parameters with tuned values.

Models	VGG16	ResNet	Wide ResNet
Hyperparameters			
Optimizer	Adam	RMSProp	Adam
Learning rate	0.0001	0.0001	0.0001
Activation function	tanh	relu	relu
Number of neurons in customized layers	128	512	256
Number of epochs	100	100	100
Batch size	64	64	32

### Performance metrics

The performance of the models must be tested once they have been constructed using some evaluation metrics for the test datasets. In this study, we have evaluated the performance with accuracy, precision, specificity, sensitivity, and Area under the ROC Curve (AUC-ROC) of the multiple CNN models developed for the classification of COVID-19. The performance measurements taken from the confusion matrix, namely True Positive (TP), True Negative (TN), False Positive (FP), and False Negative (FN) indices, are used to measure these metrics. Using these indices, the metrics Accuracy, sensitivity, precision, and specificity have been calculated respectively using Eqs. (1)–(4).

Accuracy is the number of images correctly classified as a specific class out of the total number of images in that class and given by Eq. (1).

Sensitivity (Recall or True Positive Rate) is the number of images correctly classified as a specific class out of the total number of actual images in that class and can be calculated using Eq. (2).

Precision (Positive Predictive Value) is defined as the number of images correctly classified as a specific class out of the total number of images classified as that of the class and given by Eq. (3).

F1-Score is the harmonic average of the precision and recall, that is, a weighted average of precision and recall. It is calculated as in Eq. (4).

AUC—ROC curve provides a summary of the ROC curve and is used as a performance evaluation for classification issues at different threshold settings. It measures how well a binary classification model can distinguish between classes.

1 $${\text{Accuracy }} = \left( {{\text{TP}} + {\text{TN}}} \right)/\left( {{\text{TP}} + {\text{TN}} + {\text{FP}} + {\text{FN}}} \right)$$

2 $${\text{Sensitivity }} = {\text{TP}}/\left( {{\text{TP}} + {\text{FN}}} \right)$$

3 $${\text{Precision }} = {\text{ TP}}/\left( {{\text{TP}} + {\text{FP}}} \right)$$

4 $${\text{Specificity }} = {\text{ TP}}/\left( {{\text{TP}} + {\text{FP}}} \right)$$

## Results and discussion

In this section, the performance of the proposed models is presented and compared with one another. Three CNN models are used in this study, which involve fine-tuning the models and utilizing transfer learning with LwF approaches to assess how well they perform on an unknown dataset of Delta-variant coronavirus images. The following sections discuss the results and the performance results of the pre-trained models.

### Results of proposed models

In this section, we look at how well the proposed CNN models worked. Experiments have been done with the optimally tuned hyper-parameters shown in Table 2, which gave the best results during training. Table 4 shows how well each model performs on the validation and test datasets.

**Table 4:** Performance of the proposed models.

Experiment scenario	VGG16		ResNet		Wide ResNet	
	Validation accuracy (%)	Testing accuracy (%)	Validation accuracy (%)	Testing accuracy (%)	Validation accuracy (%)	Testing accuracy (%)
Fine-tuner	95.6	96.25	97.01	97.37	97.51	98.13

**Figure 5:**
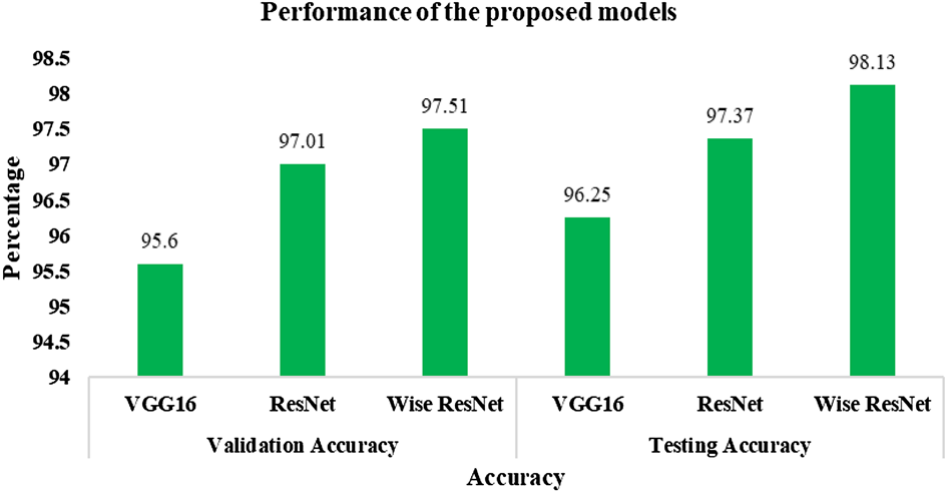
Validation versus testing accuracy of the proposed models.

A graphical visualization of the performance of the developed models is shown in Fig. 5.

Further, we have also carried out fivefold cross-validation to test the model’s ability to predict new data that was not used in training to flag problems like overfitting or selection bias and to give an insight on how the model will generalize to an independent dataset. For fivefold cross-validation, we have split the dataset into training and testing datasets with 85% and 15%, respectively. The training dataset has been subjected to fivefold cross-validation, and the results are presented in Table 5.

**Table 5:** Results of K-fold cross-validation.

Model	K-folds	Accuracy (%)	Precision (%)	Sensitivity (%)	Specificity (%)
VGG16	Fold-2	96.07	96.93	95.11	97.01
	Fold-3	96.12	96.21	95.83	96.73
	Fold-4	95.81	97.02	94.95	96.84
	Fold-5	96.02	97.41	95.46	97.21
Fivefold average		96.01	96.89	95.34	96.95
ResNet	Fold-2	97.21	97.34	97.65	97.23
	Fold-3	97.42	97.41	96.45	97.31
	Fold-4	96.79	97.34	95.92	96.89
	Fold-5	97.45	96.94	97.58	96.48
Fivefold average		97.22	97.26	96.90	96.98
Wide ResNet	Fold-2	98.21	98.07	97.52	98.46
	Fold-3	97.89	97.88	97.74	97.49
	Fold-4	97.93	98.25	98.34	97.83
	Fold-5	98.06	97.99	97.39	98.36
Fivefold average		98.02	98.05	97.75	98.04

**Figure 6:**
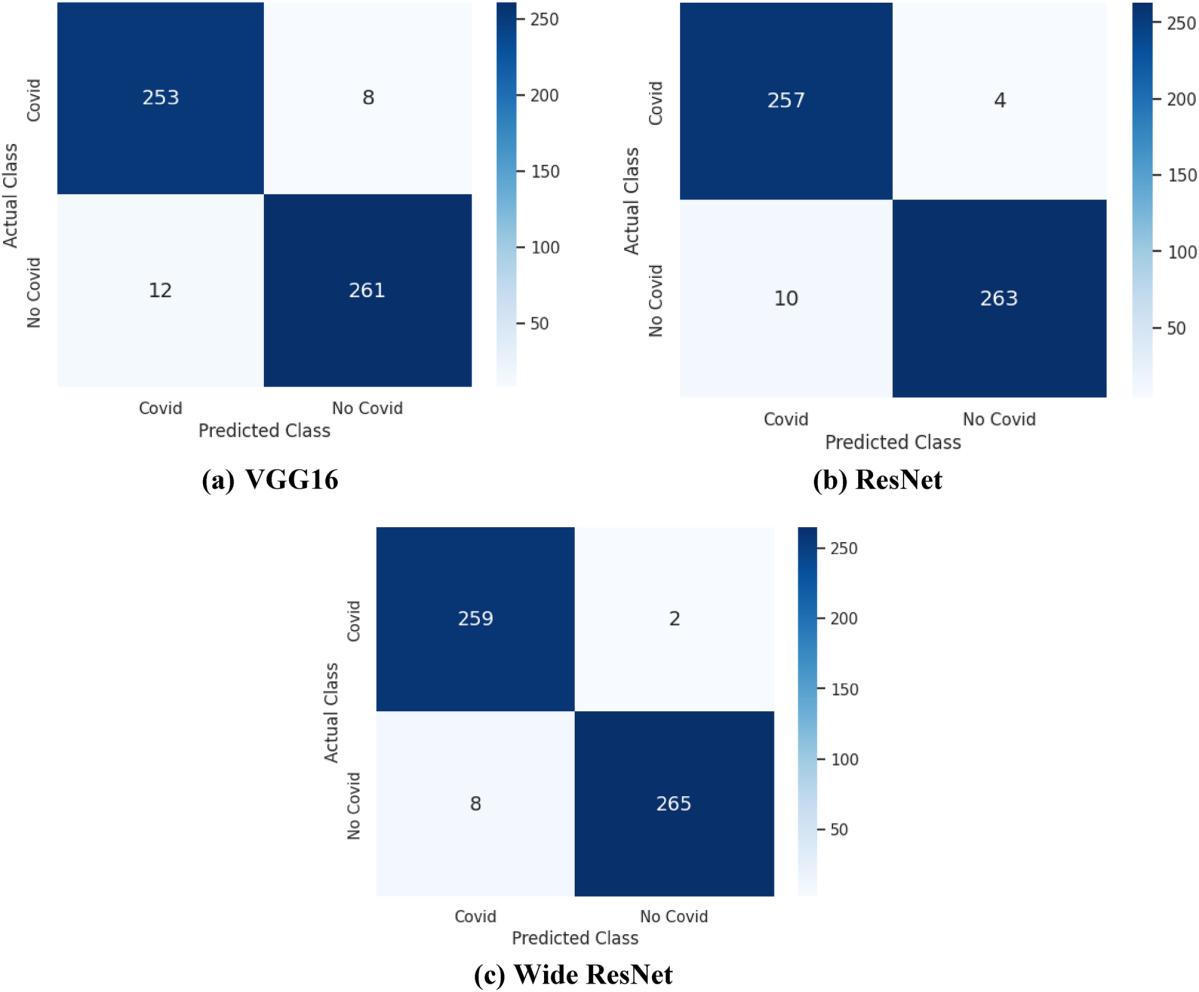
Visualization results for fine-tuned models.

We calculated the values of performance metrics, such as accuracy, precision, specificity, and sensitivity, using the indices mentioned in Section “Performance metrics”. We use the confusion matrix obtained during training and testing to determine these indexes. Figure 6 shows the confusion matrix for the set of experiments that were conducted for all models. The confusion matrix diagonals denote correct classifications. X-axis denotes the predicted classes and Y represents the actual classes. In Fig. 6a, for instance, the confusion matrix of VGG16 shows the diagnostic performance of the model in identifying COVID-19 and normal images from the dataset. Table 6 presents the performance of the pre-trained models used in this work.

**Table 6:** Performance of the pre-trained models.

Models	Accuracy (%)	Precision (%)	Sensitivity (%)	Specificity (%)
VGG16	96.25	96.93	95.47	97.03
ResNet	97.37	98.47	96.25	98.50
Wide ResNet	98.12	99.23	99.00	99.25

Table 6 shows the performance evaluation results of the pre-trained models on various performance metrics. The experimental results showed that the fine-tuning strategy did better than the feature extractor strategy regarding overall accuracy and classification performance. We achieved these results by fine-tuning the higher layers of the convolution base to learn the features specific to our dataset.

### Transfer learning over a new task using LwF

**Figure 7:**
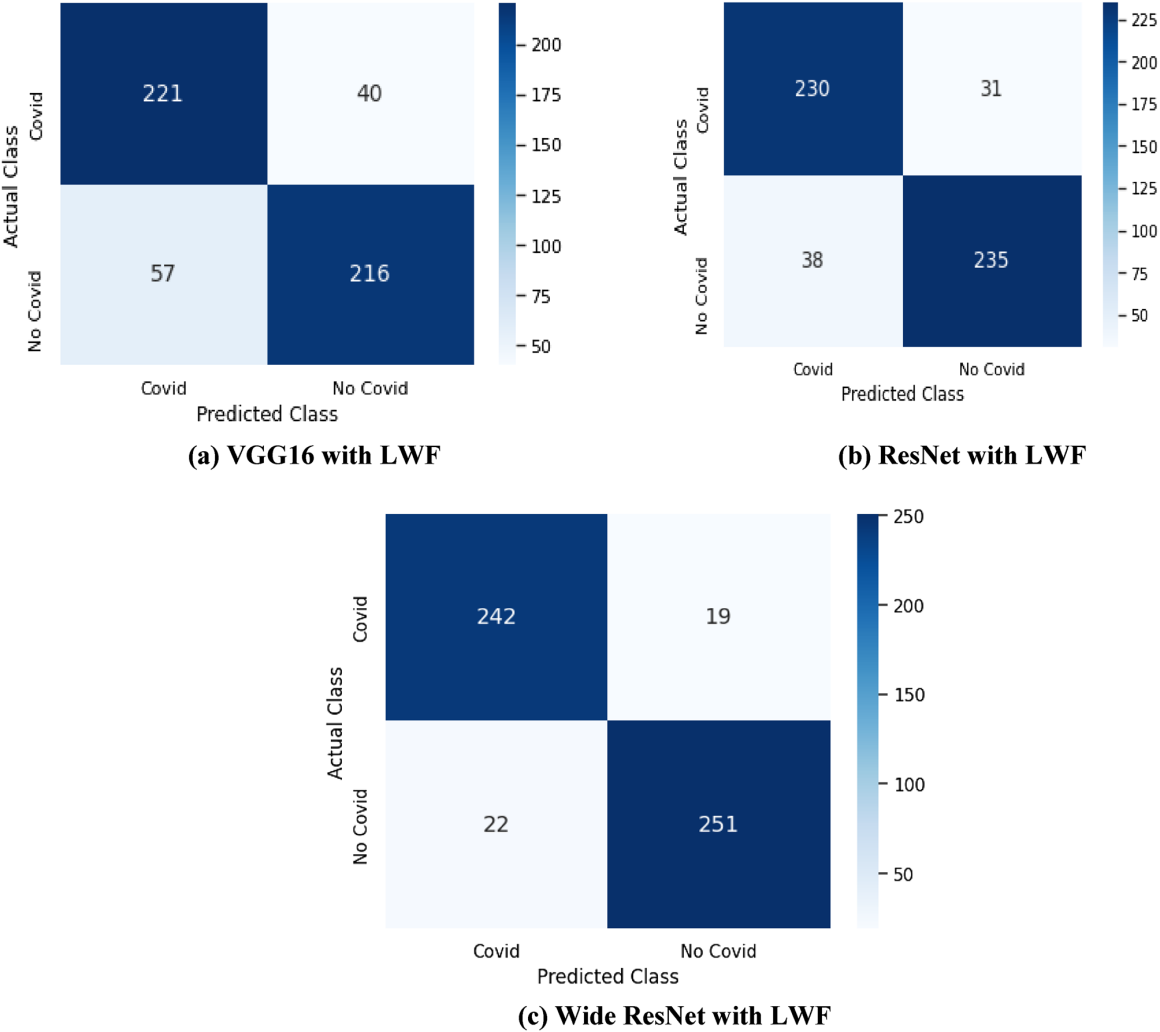
Confusion matrix for the models with LwF.

This section demonstrates how well the LwF and proposed models perform on a new set of CT images with infection from the Delta variant. The LwF only uses new images to train the network, but it keeps the original capabilities of the model. When integrating LwF with the proposed models, the shared parameters (PS) of the feature extraction layers and the task-specific parameters (PO) of the classification layers for the original dataset (which has been used for training) were kept. Still, the task-specific parameters (PB) of the new set of images have been changed. As a result, such models learned parameters that work well on original and new images. We only used sample CT images of the Delta variant for training using LwF; that is, during the retraining process, the models are trained without using the original augmented dataset. To retrain the models on the new images, we added neurons to the output layer (the sigmoid layer) and gave the weights a random initialization. To optimize the models for both the previous tasks and the new task, LwF first inserts neurons for the new task in the output layer. After that, it trains all the parameters. But, the old task set is not used in training. The quantity of recently added parameters is calculated by multiplying the number of neurons added to the output layer by the number of neurons in the previous shared layer. The added values are minimal when compared to the number of parameters in the network. The performance values for accuracy, precision, recall, and f1-score of different models combined with LwF for the classification of CT images of Delta variant samples are shown in Table 7, and the confusion matrices for these models are shown in Fig. 7.

**Table 7:** Performance of the models with LwF for the new task (Delta variants).

Models	Accuracy	Precision	Sensitivity	Specificity
VGG16	81.64	84.67	79.5	84.38
ResNet	87.08	88.12	85.82	88.35
Wide ResNet	92.32	92.72	91.67	92.96

Table 7 shows that the sensitivity of the wide ResNet model is significantly higher than that of other models. This suggests that sensitivity is more affected by the prevalence of the positive class and that wide ResNet has done an excellent job of correctly identifying CT scans with Covid infection. With high sensitivity, there are fewer false negative results, and fewer cases are overlooked.

Further, we have also calculated the AUC-ROC for the developed models with LwF, as shown in Table 9. AUC-ROC is generally preferred over accuracy because it is a much better indicator of model performance. To determine how well LwF works, we first tested the fine-tuned pre-trained models on the new CT images without LwF and then tried them with LwF. Tables 8 and 9 show the results.

**Table 8:** Performance of Fine-tuned proposed models on CT images of Delta variants and original samples without LwF.

Models	Accuracy (%)	
	Delta samples (New task)	Actual samples (Old task)
VGG16	65.73	97.19
ResNet	71.16	97.57
Wide ResNet	76.78	98.31

**Table 9:** Performance of Fine-tuned proposed models on CT images of Delta variants and augmented samples using LwF.

Models	Accuracy (%)		AUC
	Delta samples (New task)	Actual samples (Old task)	
VGG16	81.64	88.58	0.8198
ResNet	87.08	92.32	0.8722
Wide ResNet	92.32	93.08	0.9234

**Figure 8:**
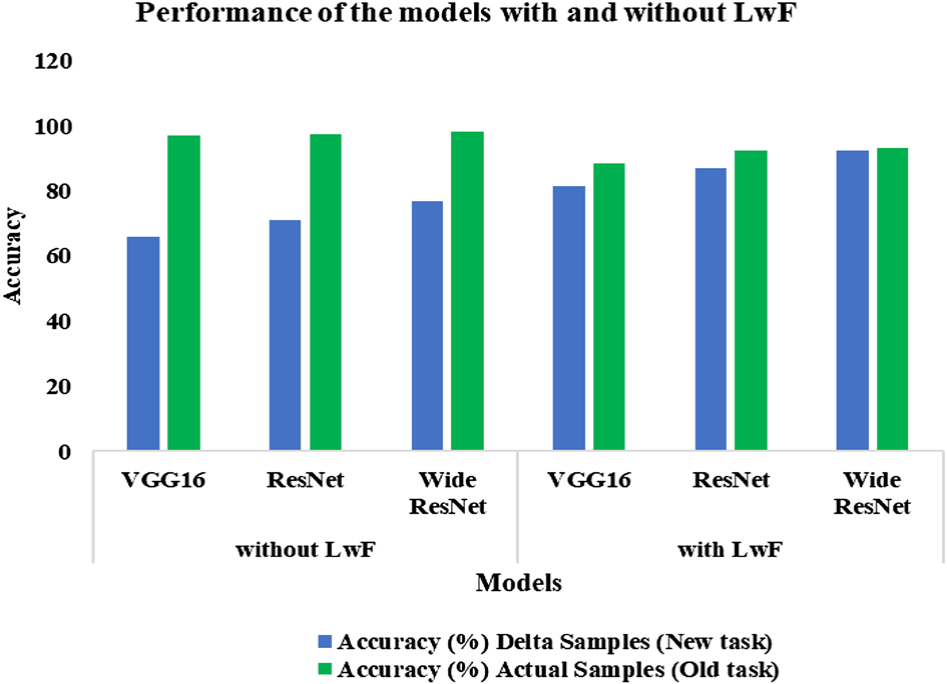
Performance of the models with and without LwF.

As seen in Table 8, the wide Resnet has achieved better accuracy than other pre-trained models. Wide ResNet outperformed other models because widening consistently enhances performance across residual networks of various depths. The wide 16-layer ResNet has a similar number of parameters and the same accuracy as a 1000-layer thin deep network. Figure 8 depicts the results of using LwF and without LwF.

## Findings and discussion

This study aims to classify CT images into two categories, Covid and Non-covid, using old and modern CNN models. The experimental results have been provided in “Results and discussion” section. In this section, we discuss the results of our investigation. The classification accuracy of the pre-trained models has been improved by fine-tuning a few top layers (Fine-tuner). The experimental findings reveal that fine-tuning the pre-trained model helps retrain the weights of a few top layers, enabling the models to acquire image-specific features and improve their accuracy. Among all proposed models, the wide ResNet exhibited the best performance. Widening consistently enhances performance across residual networks of various depths, which accounts for this performance. By widening ResNet, the network can be shallower with improved accuracy. So, wide ResNet showed better performance and higher efficiency with fewer layers.

We analyzed the model’s performance to find how well the proposed models could transfer knowledge using new CT images with stains of the coronavirus Delta variant. The experimental results were not promising in accurately identifying the COVID-19 infection caused by Delta variants. This is because the models have not been retrained on the new image set. When we tested the models again with the original images, we found that they were less accurate than before. As the number of tasks increases, storing and retraining such images becomes impossible. Furthermore, adding new capabilities to a CNN wipes out the training data for the existing capabilities. Hence, we turned to apply LwF, which retrains the network using the new images while keeping the network’s original abilities. The experimental results in “Results and discussion” section proved that LwF integration into the developed models helps classify old and new tasks. This approach outperforms widely-applied feature extraction and fine-tuning adaptation techniques, and it outperforms multitask learning that employs original task data that we presume is unavailable. But, LwF does not require original tasks. Table 10 shows how the developed models perform when integrating the LwF and fine-tuning approaches.

**Table 10:** Finetuning versus LwF.

Models	Attributes		
	Performance over		Requirement of original images to improve the performance
	New images	Original images	
Fine-tuning	Good	Poor	Yes
LwF	Good	Good	No

For an increased performance on new tasks, LwF may be possible to replace fine-tuning using similar old and new task datasets. Subsequently, the accuracy of the old task will be equivalent to that of the original network, provided the model is maintained in such a way that task-specific characteristics from previous datasets give identical outputs on all relevant images.

In this study, we implemented LwF, a multi-task learning strategy for CNNs that enables CNNs to acquire favorable performance on new tasks while maintaining comparable performance achievements on the previous tasks. The performance of the models on the new dataset shows the efficacy of LwF that has been validated, and experimental findings demonstrate the models’ superior performance with LwF over fine-tuning method.

### Error analysis

To acquire a more profound knowledge of the challenges inherent in the transfer learning process, we investigated the errors resulting from the proposed models. Images that the models improperly classified are subjected to error analysis to uncover the underlying causes of the errors. The performance measure, namely TP, FP, FN, and TN, are the possible outcomes of a classification model applied to an image dataset. For example, in Fig. 6c, we see that the true positive for the Covid-19 class is 259. This indicates that 259 out of 261 samples have been correctly classified as Covid, whereas 2 have yet to. Similarly, only 265 instances of the non-covid class have been correctly classified as not infected, while 8 examples have been misclassified as Covid infected. A few cases are provided below.

**Figure 9:**
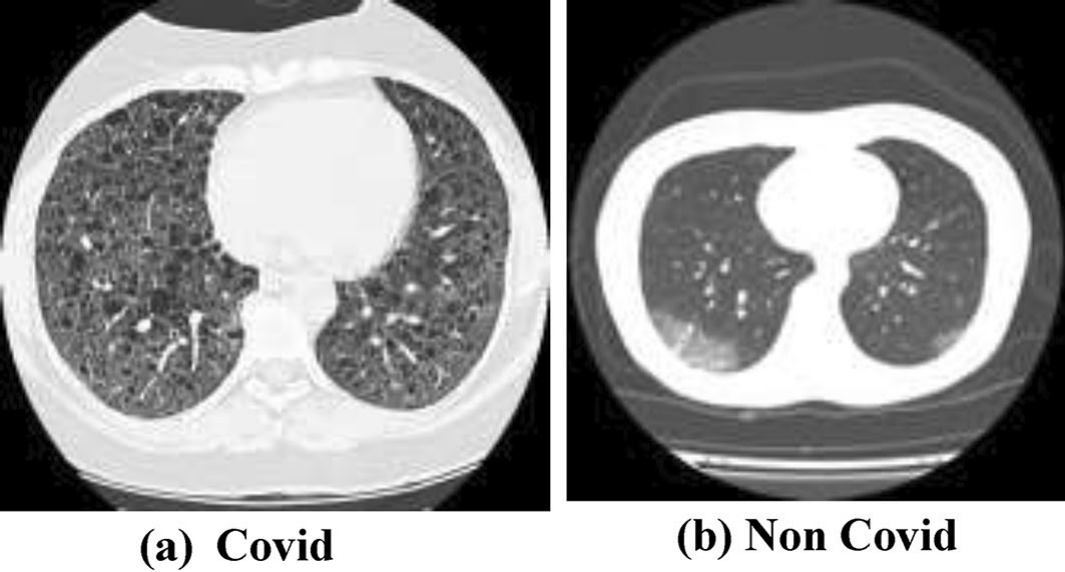
Error analysis.

Although the image in Fig. 9a belongs to Covid, VGG16 predicted it is Non-Covid. The indistinctness of the medical images, caused by the presence of lesions and tissues in CT scan pictures, made the prediction process more difficult. This misclassification can be overcome by using appropriate image pre-processing techniques. Therefore, even though the feature map shows the presence of infection, we cannot say for certain that this is the cause of misclassification. On the other hand, ResNet has correctly categorized the image. Similarly, the non-Covid image presented in Fig. 9b is misclassified as the Covid infected according to the VGG16 model. There are potential reasons for this, one of which is that the models did not successfully extract the features. Ground Glass Opacity (GGO), which refers to hazy gray patches on lung CT scans and suggests lung density, is the key feature of a CT scan that indicates infection. On a chest CT with a lung window, GGO is easily visible. However, it can be mistakenly present if the patient needs to take a deep enough breath during the CT scan. As a result, the misleading GGO images were almost always incorrectly paired with COVID-19 lesions. Even patients with minor symptoms may develop GGO in their lungs. Hence, more research is needed to examine the relevance of imaging features generated from GGO in predicting COVID-19 separately. Further, using the images that were incorrectly classified can help enhance the accuracy of the classification. Instead of analyzing both classes of images, we should concentrate on collecting data from the class that has been misclassified. This will allow us to obtain more useful information and insights to enhance the predictive accuracy of the model.

## Conclusions

This study demonstrates the application of three pre-trained deep convolution neural network models, namely VGG-16, ResNet, and wide ResNet, with fully connected layers for the detection and prediction of COVID-19 disease. The pre-trained model utilized transfer learning to improve the classification accuracy of the CNN model in predicting the SARS-CoV-2 virus using computed tomography (CT) images. The hyper-parameters of the three deep CNN models are tuned and optimized with Bayesian Optimization. The performance of the developed models is improved with a finetuning mechanism for COVID-19 disease detection. In the proposed work, we have incorporated the LwF to improve the generalization capabilities of the pre-trained models better to discriminate the normal data from an infected one when applied to a new dataset. We have evaluated the performance of the finetuned deep CNN model with the LwF method on the original dataset and a delta-variant COVID-19 dataset. The experimental results are analyzed to demonstrate the discriminative power and robustness of the deep CNN architecture with the LwF method in detecting different variants of the SARS-CoV-2 virus in the CT scan images. Of the three fine-tuned deep CNN models with the LwF method, the wide ResNet model shows superior classification performance and can discriminate and classify both original and delta-variant datasets compared with the other two models. Hence, the proposed work has the potential to be developed as the baseline model for clinical diagnostic systems to investigate further experiments on more variants of SARS-CoV-2 infections.

As an extension of the proposed work, further investigation can be conducted on the images acquired from different sources to analyze their impact on detecting COVID-19 infection. Also, our goal is to apply the proposed fine-tuned deep CNN model with LwF on other imaging modalities like chest X-rays and lung ultrasounds to detect COVID-19 disease and other diseases in clinical practice.

## Data Availability

The datasets generated and analyzed during the current study are publicly available in (i) [Covid-19 Image Dataset] repository^17^, (ii) [COVID-19 Chest X-Ray Image Repository] repository^18^, (iii) [COVID-19 Image Data Collection] repository^19^, (iv) [COVID-19 imaging datasets] repository^20^.
